# Variation in the Complex Carbohydrate Biosynthesis Loci of *Acinetobacter baumannii* Genomes

**DOI:** 10.1371/journal.pone.0062160

**Published:** 2013-04-16

**Authors:** Johanna J. Kenyon, Ruth M. Hall

**Affiliations:** School of Molecular Bioscience, The University of Sydney, Sydney, New South Wales, Australia; University of Florida, United States of America

## Abstract

Extracellular polysaccharides are major immunogenic components of the bacterial cell envelope. However, little is known about their biosynthesis in the genus *Acinetobacter*, which includes *A. baumannii*, an important nosocomial pathogen. Whether *Acinetobacter* sp. produce a capsule or a lipopolysaccharide carrying an O antigen or both is not resolved. To explore these issues, genes involved in the synthesis of complex polysaccharides were located in 10 complete *A. baumannii* genome sequences, and the function of each of their products was predicted via comparison to enzymes with a known function. The absence of a gene encoding a WaaL ligase, required to link the carbohydrate polymer to the lipid A-core oligosaccharide (lipooligosaccharide) forming lipopolysaccharide, suggests that only a capsule is produced. Nine distinct arrangements of a large capsule biosynthesis locus, designated KL1 to KL9, were found in the genomes. Three forms of a second, smaller variable locus, likely to be required for synthesis of the outer core of the lipid A-core moiety, were designated OCL1 to OCL3 and also annotated. Each K locus includes genes for capsule export as well as genes for synthesis of activated sugar precursors, and for glycosyltransfer, glycan modification and oligosaccharide repeat-unit processing. The K loci all include the export genes at one end and genes for synthesis of common sugar precursors at the other, with a highly variable region that includes the remaining genes in between. Five different capsule loci, KL2, KL6, KL7, KL8 and KL9 were detected in multiply antibiotic resistant isolates belonging to global clone 2, and two other loci, KL1 and KL4, in global clone 1. This indicates that this region is being substituted repeatedly in multiply antibiotic resistant isolates from these clones.

## Introduction

*Acinetobacter baumannii* is amongst the most troublesome Gram-negative bacterial pathogens worldwide, due to the prevalence of strains that are resistant to most antibiotics currently used. The majority of multiply antibiotic resistant clinical isolates fall into two globally disseminated clonal groups, global clone 1 (GC1) and global clone 2 (GC2) [1]. Persistence in the hospital environment, enhanced by resistance to disinfection and resistance to long periods of desiccation, contributes to the success of the organism [2]. Rather little is known about the virulence mechanisms of *A. baumannii*. However, it has been established that surface polysaccharides play a major role in pathogenesis [3], [4], and enhance motility and protect cells against complement-mediated bactericidal activity [2]. For many Gram-negative bacteria, capsule (K antigen) and/or O-antigen surface polysaccharides are major virulence determinants that are highly immunogenic. Moreover, the composition and structure of both K and O antigens can vary considerably between different strains of the same species [5], [6].

K and O antigens both extend from the cell surface of Gram-negative bacteria as variable-length polymers composed of oligosaccharide units called repeat units [7], [8]. The fundamental difference between capsules and O antigens is that capsular polysaccharide is exported directly to the cell surface whereas the O antigen is the outer-most constituent of lipopolysaccharide (LPS), a multi-component structure that is formed in the periplasm prior to export [5]. An O-antigen ligase, WaaL, transfers the polymer of repeat units (the O antigen) to the lipid A-core moiety, commonly referred to as the lipooligosaccharide (LOS), to form the LPS. In the absence of a WaaL ligase, LOS includes a lipid A outer membrane anchor attached to a core oligosaccharide that consists of an inner core and an outer core (OC). When LPS cannot form, the undecorated LOS is exported to the cell surface.

**Figure 1:**
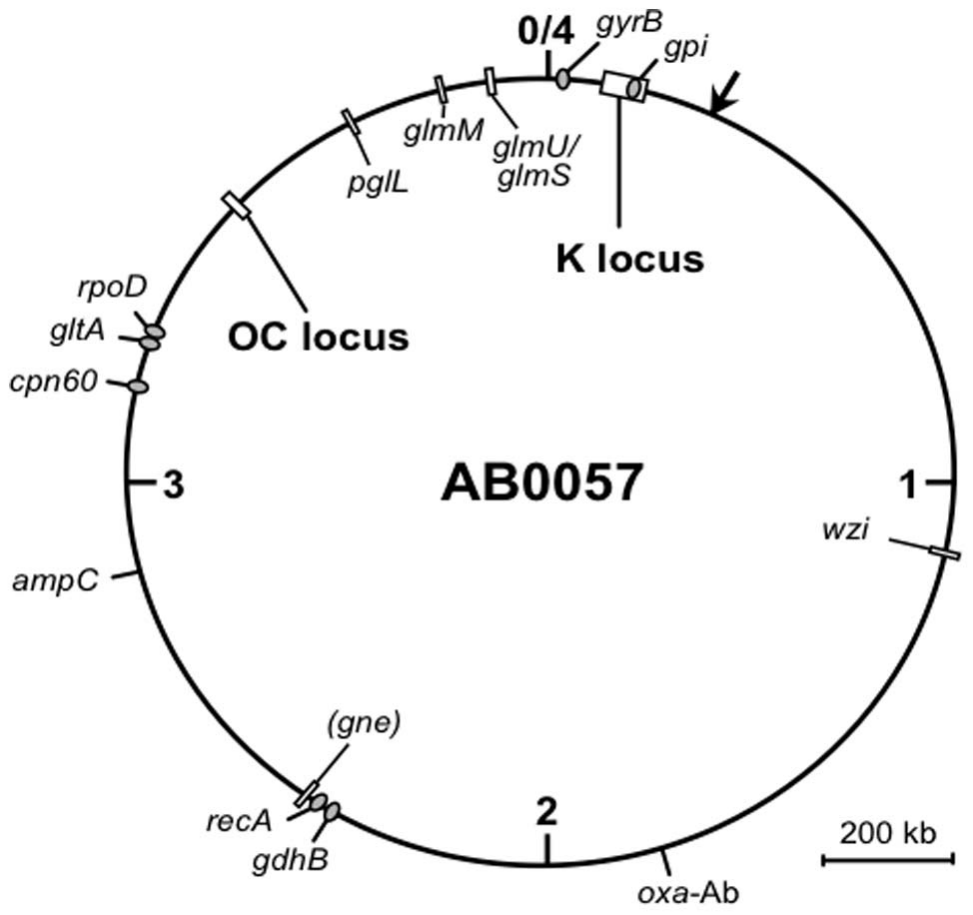
Map of the genome of GC1 isolate AB0057 showing positions of genes and loci. The genes and loci described in this study are indicated by boxes, with their names shown inside the circle. Locations of genes included in the Oxford MLST scheme are indicated by the ovals with names outside the circle. The *gpi* gene is within the K locus. The arrow indicates the *comM* gene, the location at which transposons carrying resistance to antibiotics are often inserted. The numbers represent megabases from origin, and a scale bar is shown. The AB0057 sequence was chosen as the representative genome [GenBank accession CP001182] because it begins at the origin of replication (0/4) and this position differs in other genomes. Genes outside the K and OC loci that code for PglL, GlmS, GlmM, GlmU, Gne, and Wzi are found in the at locus tags AB57_3633, AB57_3845, AB57_3769, AB57_3844, AB57_2335, and AB57_1078 respectively.

Currently, there is confusion in the published literature about whether *A. baumannii* produces a capsule or an O antigen or both [9]. This partly arises from the fact that the repeat units of capsules and O antigens can have similar structures and are synthesised by similar enzymatic pathways. Several different repeat unit structures, reported as either capsule or O antigen, have been solved for *A. baumannii* ([10]; and reviewed in [9]) or other *Acinetobacter* species [11]–[17]. A group of genes associated with the synthesis of a complex carbohydrate polymer have been described as O-antigen gene clusters in several *A. baumannii* genomes [18]–[21]. The location of these clusters is conserved (see Figure 1) but the locus exhibits extensive diversity of sequence and gene content. However, the equivalent region in the *Acinetobacter baylyi* ADP1 (previously *A. calcoaceticus* BD413) genome was described as the capsule gene cluster because it contains all the genes required for the synthesis of the known ADP1 capsule repeat unit [11], and a gene encoding a WaaL homologue was not found [22]. More recently, the absence of a gene for a WaaL ligase in *A. baumannii* strain ATCC 17978 has also been noted [4]. In other cases, a WaaL homologue was not specifically sought.

Synthesis of both capsular and O-antigen polysaccharides begins with the synthesis of the repeat unit in the cytoplasm. A pre-formed sugar precursor is transferred to the inner membrane lipid carrier, undecaprenol phosphate (UndP), by an initiating transferase. Substrate-specific glycosyltransferases then sequentially add further sugars via specific linkages to form a repeat unit containing a few sugars. This unit is then translocated across the inner membrane into the periplasm where they are polymerised into a chain. The genes required for the synthesis of capsule or O antigen are generally clustered and variation in these loci gives rise to structural heterogeneity in K and O antigens. In *Acinetobacter* species, this locus also includes genes, *wza*, *wzb* and *wzc,* that encode proteins related to those involved in the export of capsule in other bacteria [23] and this is consistent with the conclusion that a capsule is synthesised. In the GC1 *A. baumannii* strain 307-0294, transposon knockouts of *wza* and *wzc* prevented expression of a surface polysaccharide [3], which has been shown to be an immunogenic capsule and named K1[24]. These genes have also been identified in the related species, *Acinetobacter venetianus* RAG-1 (previously *A. lwoffii* RAG-1), in a gene cluster that has been shown to be required for the production of the capsule (also known as emulsan; reviewed in [25]).

In Gram-negative bacteria, the OC component of the LOS is also generally variable, and the genes required for OC synthesis are clustered but located at a different position on the chromosome to the O-antigen or capsule gene clusters [26]. The OC locus usually includes the *waaL* gene that encodes the O-antigen ligase [6]. For *A. baumannii*, 2 different OC structures have been reported [27], [28], one of which has been found in a second strain [29]. In the genomes, a second variable region containing polysaccharide genes, also described as an O-antigen gene cluster [21], is likely to represent the OC locus (see Figure 1).

Because of the clinical significance of *A. baumannii* and particularly multiply antibiotic resistant isolates, several genome sequences, mostly from members in the two major clonal complexes, have been completed (see Table 1). Many more genomes are available as drafts. In this study, we have used the completed genome sequences to examine whether a K or O antigen is likely to be produced, and to describe in detail the variable regions associated with the synthesis and processing of complex surface polysaccharides.

**Table 1:** *A. baumannii* completed genome sequences^a^.

Strain	GC^b^	GenBank accession numbers	Reference
AYE	1	CU459141	[68]
307-0294	1	CP001172	[18]
ACICU	2	CP000863	[19]
ATCC 17978	-	CP000521	[69]
AB0057	1	CP001182	[18]
SDF	-	NC_010400	[68]
1656-2	2	CP001921	[70]
TCDC-AB0715	2	NC_017387	[71]
MDR-ZJ06	2	CP001937	[72]
MDR-TJ	2	NC_017847	[73]

a As of 30^th^ September, 2012.

b GC: Global clone.

## Results

### O antigen or LOS and capsule?

The location of the two gene clusters involved in the synthesis of complex polysaccharides in the 10 completed *A. baumannii* genome sequences available in GenBank (Table 1) and their properties are listed in Table 2. The larger locus has been identified in several studies [18]–[21], while part of the smaller region was described only by Di Nocera et al. (2011). The positions of these two loci relative to the origin of replication (in the genome of GC1 isolate AB0057 [GenBank accession CP001182]) are shown in Figure 1.

**Table 2:** Location of K and OC loci in completed genomes.

		K locus					OC locus				
					Position of flanking genes^b^					Position of flanking genes^c^	
Strain	GC^a^	Name	Size (kb)	No. ORFs	*fkpA* start	*lldP* start	Name	Size (kb)	No. ORFs	*ilvE* stop	*aspS* stop
AYE	1	KL1	22.58	18	3860319	3837737	OCL1	8.78	9	586244	595022
307-0294	1	KL1	22.58	18	3690644	3668062	OCL1	8.78	9	584973	593752
ACICU	2	KL2	24.77	22	77817	102585	OCL1	8.78	9	3355679	3346901
ATCC 17978	-	KL3	23.08	20^d^	56834	79909	OCL2	8.79	9	3356337	3347548
AB0057	1	KL4	28.60	23^d^	93756	122359	OCL3	8.91	9	3516588	3507674
SDF	-	KL5	25.51	22	62443	87951	OCL1a	9.40	9	484334	493730
1656-2	2	KL6a	24.61	21	88096	112706	OCL1	8.78	9	3399651	3390872
TCDC-AB0715	2	KL7	25.65	22	80129	105781	OCL1	8.78	9	3610272	3601494
MDR-ZJ06	2	KL8	32.78	27^d^	77157	109935	OCL1	8.78	9	3442833	3434054
MDR-TJ	2	KL9	25.46	21	3894956	3869492	OCL1	8.78	9	558089	566867

a GC: Global clone.

b *fkpA* and *lldP* are divergently transcribed, with start codons closest to the K locus.

c *aspS* and *ilvE* are transcribed in opposite directions, with stop codons closest to the OC locus.

d Some ORFs with potential frameshifts (see Figure 3).

**Figure 2:**
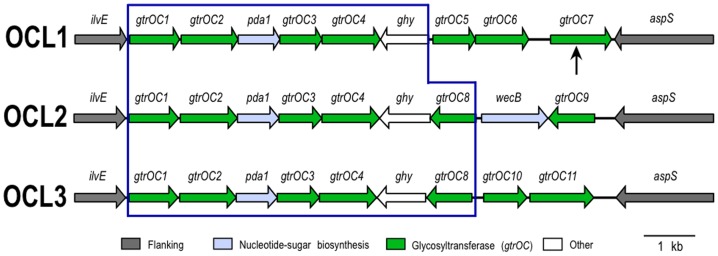
Genetic arrangement of OC loci in *A. baumannii.* OC-locus names are indicated on the left. Horizontal arrows represent genes showing the direction of transcription, with gene names above. Flanking genes are shown in grey, and other genes are coloured by the predicted functional group of their product with the colour scheme shown below. The vertical arrow indicates the position of ISAba7 in OCL1a (strain SDF). The module that is conserved is boxed in blue. The figure is drawn to scale from the GenBank entries listed in Table 1. Gene annotations: *gtrOC* is OC glycosyltransferase, *ghy* is glycosyl hydrolase, *pda* is polysaccharide deacetylase, and *wecB* encodes a UDP-D-Glc*p*NAc 2-epimerase.

The smaller locus is located between *ilvE* and *aspS* genes [locus tags AB57_3409 and AB57_3398 in CP001182] that are unlikely to be involved in polysaccharide biosynthesis as they encode a branched-chain aminotransferase and a tRNA aspartate synthetase, respectively. Three forms of this region were found in the genomes examined here (Figure 2), and each includes genes for sugar biosynthesis and glycosyltransfer, but none for repeat unit processing. This locus is likely to be responsible for the synthesis of the OC region of the LOS and will be referred to as the OC locus.

**Figure 3:**
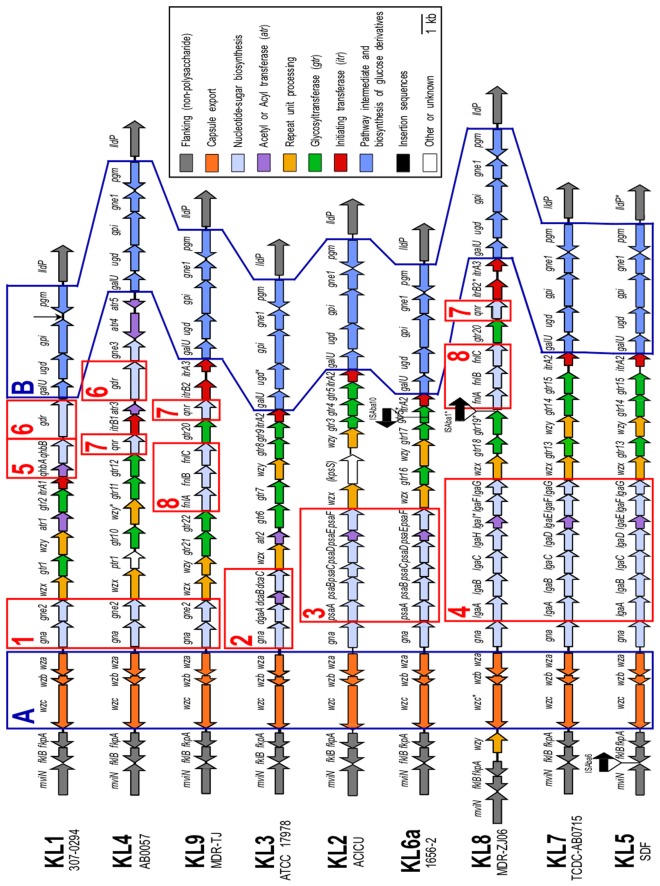
Arrangement of K loci in *A. baumannii* completed genomes. K-locus names with strain names beneath are indicated on the left. Horizontal arrows represent genes showing the direction of transcription, with assigned gene names shown above. * indicates internal frameshifts. Genes are coloured by the predicted functional group of their gene product with the colour scheme shown on the right. Modules that are shared by all are boxed in blue and labelled A and B. Modules predicted to code for enzymes required for synthesis of the same sugar are boxed in red and numbered. The black vertical arrow indicates the absence of *gne1* in KL1. The figure is drawn to scale from the GenBank entries listed in Table 1, and the positions of the loci in the completed genomes are in Table 2. Gene annotations are defined in Table 3.

The larger locus is located between *fkpA* and *lldP* [locus tags AB57_0090 and AB57_0116 in CP001182], which encode a peptidyl-prolyl *cis-trans* isomerase and an L-lactate permease respectively, that would not contribute to polysaccharide synthesis. Nine types were found in the 10 genomes assessed (Figure 3); AYE and 307-0294 have identical sequences in this region [18]. Each K locus contains genes that code for enzymes involved in the synthesis and assembly of the oligosaccharide repeat units found in O antigens or capsules, and for repeat unit translocation across the inner membrane (Wzx) and subsequent polymerisation (Wzy). These genes are typically found together in capsule and O-antigen biosynthesis loci in many Gram-negative bacterial species. Each locus also contains polysaccharide export genes (*wza, wzb*, and *wzc*), which, in *E. coli*, are only associated with the export of capsular polysaccharides [5].

A *waaL* gene, encoding the ligase required for addition of the carbohydrate polymer (O antigen) to lipid A-core, which is usually in the OC locus [6], was not found in any of the *A. baumannii* OC loci (see Figure 2). All proteins predicted from the genome sequences listed in Table 1 were examined for homology to WaaL from *P. aeruginosa* PAO1 and the five defined types of WaaL from *E. coli* (see Methods). Only the ATCC 17978 PglL *O*-oligosaccharyltransferase [GenPept accession YP_001086175.1] that has been shown to be involved in a protein *O*-glycosylation system [4], and the equivalent gene in other genomes was found. The location of the *pglL* gene [locus tag AB57_3633 in CP001182] is shown in Figure 1. In all 10 genomes, the gene that codes for the PglL homologue and the PglL protein are incorrectly annotated (Lipid A core--O-antigen ligase, O-antigen polymerase family protein, or hypothetical protein) (see Table S1). For most species, PglL has strict specificity for protein substrates [30]. Hence, although PglL mediated ligation of polysaccharide to lipid A-core has not been conclusively excluded, it was concluded that *A. baumannii* does not produce an O antigen. The larger locus is therefore likely to be responsible for the synthesis and export of a capsular polysaccharide, and is hereafter referred to as the K locus.

### Variation in the OC locus

The three forms of the OC locus identified were assigned numbers to differentiate them. Each consists of a conserved portion (boxed in blue in Figure 2) followed by a variable region. The most common arrangement is OCL1, found in 8 of the 10 genomes (see Table 2), and the OCL1 DNA sequences share pairwise identities greater than 98%. However, the SDF OCL1 locus (designated OCL1a) includes an ISAba7 insertion sequence (IS) that disrupts a glycosyltransferase gene (indicated by the vertical arrow in Figure 2). Single representatives of OCL2 and OCL3 are found in the ATCC 17978 and AB0057 genomes, respectively. However, they both share more than 97% identity with OCL1 in the region boxed in Figure 2. This conserved region extends further in OCL2 and OCL3 to include an additional gene.

For the majority of proteins encoded by genes in the OC loci, BLASTp matches were poor though many matches were to LOS biosynthesis proteins of other species. The gene annotations shown in Figure 2 were largely based on protein family (Pfam) predications. OCL1 and OCL3 include 7 genes (*gtrOC*) for putative glycosyltransferases, whereas OCL2 has 6. The structure of the LOS of ATCC 19606 has been solved [28], and the OC component would require 7 glycosyltransferases. Consistent with this, it was found that the draft genome of ATCC 19606 [GenBank accession NZ_GG704577.1] carries OCL1. Most of the sugars included in OCL1 could be synthesised by proteins encoded by genes in module B of the capsule gene cluster (see below). However, the ATCC 19606 OC oligosaccharide also includes D-glucosamine (Glc*p*N) and D-galactosamine (Gal*p*N) side groups, and the *pda* gene encodes the deacetylase, Pda, which could convert UDP-*N*-acetyl-D-glucosamine (UDP-D-Glc*p*NAc) to Glc*p*N, and UDP-*N*-acetyl-D-galactosamine (UDP-D-Gal*p*NAc) to Gal*p*N. A role for the predicted Ghy hydrolase (*ghy* gene in Figure 2) could not be predicted from the structure. The *wecB* gene in OCL2 predicts a protein with 57% identity to a UDP-D-Glc*p*NAc 2-epimerase, WecB (previously known as RffE), from *Psychrobacter sp*. PAMC21119 [GenPept accession ZP_10790560.1] that converts UDP-D-Glc*p*NAc to UDP-*N*-acetyl-D-mannosamine [31].

### Variation in the K locus

For simplicity, each of the 9 K locus types was assigned a number. K1 had previously been used for the 307-0294 capsule [3], and KL1 was used for the locus in GC1 isolates AYE and 307-0294. KL2 was assigned to ACICU, the first completed GC2 genome [19], and the other loci were assigned KL numbers in order of the genome publication date. When IS elements were present, letters were added after the KL number. For example, an ISAba10 disrupts a glycosyltransferase gene in the KL6a cluster of 1656-2; the uninterrupted KL6 was found in the draft genome of strain ABNIH3 [GenBank accession AFTB01000052]. All genes identified in KL1-KL9 were named according to the annotation scheme developed for this study (see Methods and Table 3). KL3, KL4 and KL8 each contained genes with frameshifts (marked with an asterisk in Figure 3). These regions in ATCC 17978 (KL3) and AB0057 (KL4) were re-sequenced and the frameshifts were found to be the result of sequencing errors. MDR-ZJ06 (KL8) was not available for re-sequencing but may also contain errors. For this genome, confirmation of the unusual placement of the *wzy* gene (see Figure 3) is also needed.

**Table 3:** Key to gene names used.

Gene name	Predicted reaction product	Predicted protein
*atr*	-	Acyl- or Acetyl- transferase
*dga*	UDP-2,3-diacetamido-2,3-dideoxy-α-D-glucuronic acid	Multiple
*fnl*	UDP-L-Fuc*p*NAc	Multiple
*galE*	UDP-D-Gal*p*	UDP-D-Glc*p* C4 epimerase
*galU*	UDP-D-Glc*p*	UTP-glucose-1-phosphate uridylyltransferase
*gdr*	UDP-4-keto-6-deoxy-D-Glc*p*NAc	UDP-Glc*p*NAc 4,6-dehydratase
*ghy* a	-	Glycosylhydrolase
*gpi*	L-Fructose-6-phosphate	glucose-6-phosphate isomerase
*gtr*	-	Glycosyltransferase
*gtrOC* a	-	Glycosyltransferase (outer core)
*itr*	-	Initiating transferase
*lga*	Legionaminic acid derivative	Multiple
*pda* a	UDP-D-GlcN	Polysaccharide deacetylase
*pgm*	D-Glucose-1-phosphate	Phosphoglucomutase
*psa*	Pseudaminic acid derivative	Multiple
*ptr*	-	Pyruvyl transferase
*qhb*	UDP-D-Qui*p*NAc4NHb	Multiple
*qnr*	UDP-D-Qui*p* NAc	UDP-6-deoxy-4-keto-D-Glc*p*NAc 4-reductase
*ugd*	UDP-D-Glc*p*A	UDP-D-Glc*p* dehydrogenase
*wecB*	UDP-D-Man*p*NAc	UDP-D-Glc*p*NAc C2 epimerase
*wza*	-	Outer membrane protein
*wzb*	-	Protein tyrosine phosphatase
*wzc*	-	Protein tyrosine kinase
*wzx*	-	Repeat unit translocase
*wzy*	-	Repeat unit polymerase

a Genes in the OC loci.

Though there are substantial differences in the composition of the K loci (Figure 3), regions of high identity between one or more loci are also seen. For example, KL5 in SDF and KL7 in TCDC-AB0715 are more than 98% identical over 23.2 kb of the 25.6 kb locus, but differ over a 1.9 kb segment. This leads to a change in two centrally located genes. The glycosyltransferases designated Gtr12 (*gtr12* in Figure 3) share only 85.9% amino acid (aa) identity, and the adjacent *wzy* genes predict proteins that are not significantly related.

Comparison of the 9 K loci revealed two relatively conserved gene modules labelled A and B in Figure 3. Module A includes the capsule export genes, *wza, wzb* and *wzc*, and module B includes genes that are involved in the synthesis of simple UDP-linked sugar precursors. However, module B of KL1 lacks the *gne1* gene. Between modules A and B lies a region containing a variable number of Open Reading Frames (ORFs) whose products are predicted to be involved sugar synthesis (regions 1 to 8 in Figure 3), glycosyltransfer (*gtr* and *itr*), glycan modification via acetylation or acylation (*atr*), and repeat unit processing (*wzx* and *wzy*).

### Module A - Genes for capsule export

The *wza, wzb,* and *wzc* genes in module A are divergently transcribed from the remainder of the locus. The flanking *fkpA* gene is highly conserved in these genomes, exhibiting pairwise DNA identities from 97.1% to 99.2%. However, there is a sharp decrease to 83–93% identity for most module A pairs. The *wza* and *wzc* genes in the KL1 locus of *A. baumannii* strain 307-0294 have been shown to be essential for the expression of the capsule on the cell surface [3]. The Wza, Wzb and Wzc proteins are also related to proteins essential for the export of *E. coli* Group 1 and 4 capsules, with pairwise identities ranging between 30.1% and 38.1%. The Wza, Wzb and Wzc proteins are believed to form a multi-protein complex that creates a channel between the inner and outer membranes to transport the capsule from the periplasm to the cell surface [5].

### Module B - Genes for the synthesis of simple UDP-linked sugars and sugar precursors

**Figure 4:**
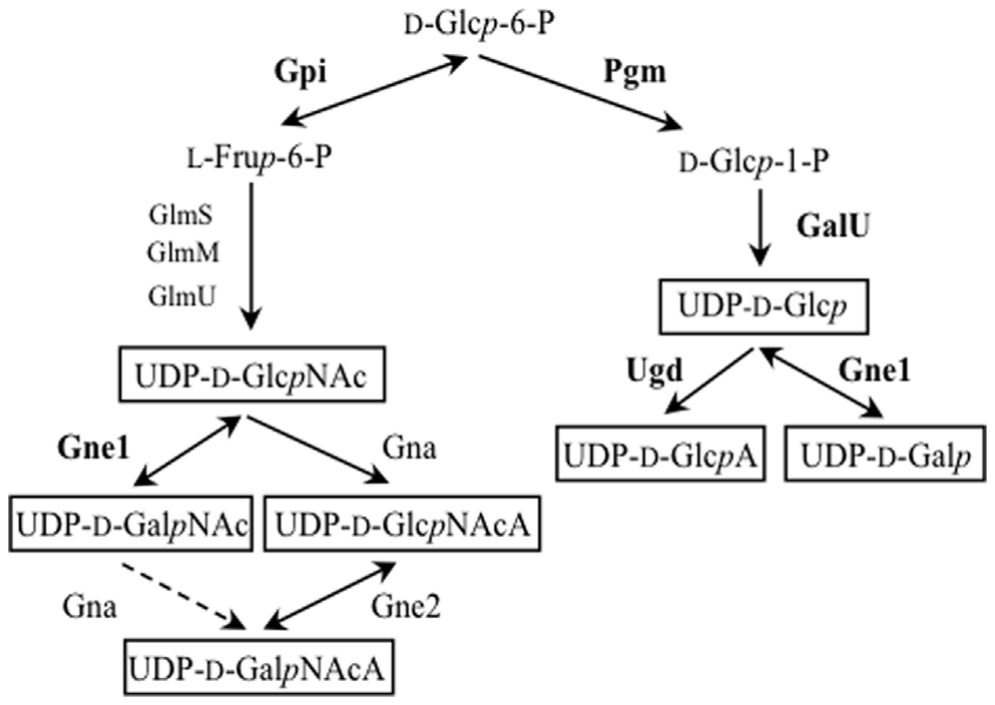
Pathways for biosynthesis of common UDP-linked sugar precursors. Common UDP-linked sugar precursors derived from glucose-6-phophate are boxed. Arrows denote the direction of each reaction, and the enzyme name is adjacent. Enzymes highlighted in bold are encoded by genes in module B (Figure 3). Abbreviations: UDP-D-Glc*p*NAc is UDP-*N*-acetyl-D-glucosamine; UDP-D-Glc*p* is UDP-D-glucose; UDP-D-Glc*p*A is UDP-D-glucuronic acid; UDP-D-Gal*p* is UDP-D-galactose; D-Glc*p*-6-P is glucose-6-phosphate; L-Fru*p*-6-P is fructose-6-phosphate; D-Glc*p*-1-P is glucose-1-phosphate; UDP-D-Gal*p*NAc is UDP-*N*-acetyl-D-galactosamine; UDP-D-Glc*p*NAcA is UDP-*N*-acetyl-D-glucosaminuronic acid; UDP-D-Gal*p*NAcA is UDP-*N*-acetyl-D-galactosaminuronic acid.

Many complex carbohydrates are derived from simple UDP-linked sugars that are typically also involved in other essential cellular metabolic pathways. Because of this, the genes required for their synthesis are commonly found outside capsule and O-antigen gene clusters [32]. However, in the *A. baumannii* genomes, module B of the capsule locus harbours several genes involved in the synthesis of key UDP-linked sugars, UDP-D-glucose (UDP-D-Glc*p*), UDP-D-galactose (UDP-D-Gal*p*), UDP-D-glucuronic acid (UDP-D-Glc*p*A), UDP-D-Glc*p*NAc, and UDP-D-Gal*p*NAc (boxed in Figure 4). Pairwise DNA identities for *lldP*, the flanking gene adjacent to module B of the K locus, range between 96.2 and 99.2% and this level of identity extends into module B (average of 90.4–98.6%) with DNA sequence identity decreasing closer to the variable region of each locus. This suggests that at least the outermost portion of module B (closest to *lldP*) may be conserved at close to housekeeping levels, and one of these genes, *gpi,* is currently used in an MLST typing scheme [33].

The reversible conversion of UDP-D-Glc*p* to UDP-D-Gal*p*, UDP-D-Glc*p*NAc to UDP-D-Gal*p*NAc, and UDP-*N*-acetyl-D-glucosaminuronic acid (UDP-D-Glc*p*NAcA) to UDP-*N*-acetyl-D-galactosaminuronic acid (UDP-D-Gal*p*NAcA) all require a C4 epimerase, and three different groups of C4 epimerases, designated GalE or Gne, have been defined [34], [35]. The Gne encoded by *gne1* in module B falls into the group that recognises UDP-D-Glc*p*/UDP-D-Gal*p* and UDP-D-Glc*p*NAc/UDP-D-Gal*p*NAc, consistent with the fact that the majority of known *A. baumannii* repeat-unit structures include D-Gal*p*, D-Gal*p*NAc, or both (see Table S2).

### Other genes for the synthesis of simple UDP-linked sugars

An alternate C4-epimerase gene, *gne2,* is found in region 1 of KL1, KL4 and KL9 (see Figure 3), and the three Gne2 proteins are close relatives, sharing pairwise identities of 71 to 90%. Gne1 and Gne2 of KL4 are only 30.3% identical to one another, but Gne2 is 73.2% identical to WbpP from *Pseudomonas aeruginosa* O6 [GenPept accession number AAF23998.1]. WbpP is a Gne-type epimerase that is known to preferentially interconvert UDP-D-Glc*p*NAcA and UDP-D-Gal*p*NAcA, but to also interconvert UDP-D-Glc*p*NAc and UDP-D-Gal*p*NAc [35]. In the case of K1, *gne1* is missing from module B, but due to the predicted substrate differences between the Gne proteins, *gne2* probably does not substitute for it. UDP-D-Gal*p* is not present in the K1 capsule repeat unit [24] and may not be produced. KL4 has a third *gne* gene (*gne3*) in the central variable region, and Gne1 and Gne3 are 90.5% identical, indicating that they may have the same or similar substrate specificity.

Gna enzymes are dehydrogenases that convert UDP-D-Glc*p*NAc to UDP-D-Glc*p*NAcA or UDP-D-Gal*p*NAc to UDP-D-Gal*p*NAcA (Figure 4), and a *gna* gene is found next to *wzc* in each K locus. Gna of KL1, KL4 and KL9 are closely related to one another (pairwise identities 87.5 to 93.4%), and are each approximately 75% identical to WbpO, the Gna equivalent from *P. aeruginosa* O6 [GenPept accession number AAF23997.1]. WbpO preferentially converts UDP-D-Glc*p*NAc to UDP-D-Glc*p*NAcA [35]. Gna from KL2, KL5, KL6, KL7, and KL8 are close relatives of the KL1, KL4 and KL9 Gna proteins, sharing on average 80% sequence identity with KL1 Gna, and 70% identity with WbpO. The Gna homologue of KL3 is 67% identical to the KL1 Gna, but is predicted to convert UDP-D-Glc*p*NAc to UDP-D-Glc*p*NAcA as the first step of the pathway for the synthesis of a complex sugar as described below. Hence, the substrate preferences of the *A. baumannii* Gna enzymes will require experimental characterization.

Additional genes involved in simple UDP-linked sugar synthesis are also found elsewhere in the *A. baumannii* chromosome (see Figure 1). The *glmS*, *glmM* and *glmU* genes [locus tags AB57_3845, AB57_3769, and AB57_3844 respectively in CP001182] are required for the synthesis of UDP-D-Glc*p*NAc. Another gene [locus tag AB57_2335 in CP001182] predicts a protein with 36% identity to Gne from *Vibrio vulnificus* YJ016 [GenPept accession: NP_935819.1], a member of the same group as Gne1 [36]. The role of this Gne remains to be established.

### KL3 in ATCC 17978

ATCC 17978 was the only strain listed in Table 1 for which there was a resolved oligosaccharide structure when this work was completed. This pentasaccharide, which has been shown to decorate several proteins via *O*-glycosylation [4], includes four simple sugars D-Glc*p*, D-Glc*p*NAc, D-Gal*p*, and D-Gal*p*NAc, as well as an *O-*acetylated derivative of 2,3-diacetamido-2,3-dideoxy-α-D-glucuronic acid (D-Glc*p*NAc3NAcA). The D-Gal*p*NAc residue is the first sugar of the structure. The same structure has also been found as the capsular polysaccharide of *A. baumannii* strain SMAL, though a sequence is not available for this isolate [10].

**Figure 5:**
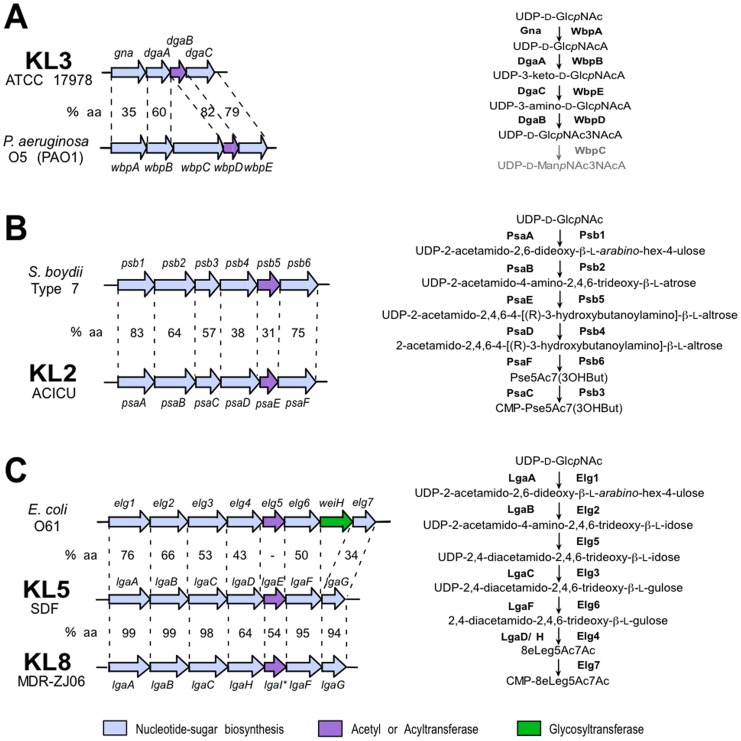
Comparison of nucleotide-linked sugar regions to related gene modules and sugar pathways. A. KL3 region 2 compared to a region from O5 from *P. aeruginosa* PAO1 [GenBank accession U50396]. The biosynthesis pathway for UDP-Glc*p*NAc3NAcA determined by [37] is shown on the right. **B**. KL2 region 3 compared to a region from the *S. boydii* Type 7 O-antigen locus [GenBank accession EU296411]. The biosynthesis pathway for CMP-Pse5Ac7(3OHBut) predicted by [39] is on the right. **C**. KL5 region 4 compared to KL8 region 4 and a region from the *E. coli* O61 O-antigen cluster [GenBank accession GU220362]. The predicted biosynthesis pathway for CMP-8eLeg5Ac7Ac [64] is shown on the right. Horizontal arrows represent genes showing the direction of transcription, with gene names shown above. Genes are coloured by the predicted function of their gene product and the colour scheme is shown the below. * indicates frameshifts. The% identity between predicted gene products is shown between aligned pairs of gene clusters. Regions are defined in Figure 3. The figure is drawn to scale from the GenBank entries listed in Table 1 and above.

The four simple sugars can be formed via pathways described in Figure 4, and the genes required for the synthesis of UDP-D-Glc*p*NAc3NAcA and construction of the oligosaccharide are also found in the KL3 locus. Immediately downstream of *gna*, a group of genes named *dga* for 2,3-diacetamido-2,3-dideoxy-α-D-glucuronic acid (region 2 in Figure 3) encode products that share significant identity with proteins from the O5 O-antigen locus in *P. aeruginosa* PAO1 (see Figure 5A). WbpB, WbpD and WbpE synthesize UDP-D-Glc*p*NAc3NAcA [37]. Though the Gna is only 35% identical to WbpA, it would substitute for it. The KL3 and PAO1 genes are in a similar order although a homologue of *wbpC*, which extends the synthesis pathway for O5 (see Figure 5A), is missing from KL3. A predicted acetyltransferase encoded by the *atr2* gene in KL3 would catalyse the final acetylation step to form UDP-D-Glc*p*NAc3NAcA4OAc from UDP-D-Glc*p*NAc3NAcA.

Genes for the four glycosyltransferases required to form the four internal glycosyl linkages of the ATCC 17978 pentasaccharide are present in KL3. The *itrA2* gene for an initiating transferase that would link the first sugar, UDP-D-Gal*p*NAc, to the UndP lipid carrier, completes the requirements for repeat-unit construction. A *wzx* gene for the translocase that moves the oligosaccharide into the periplasm, and a *wzy* gene to polymerize repeat units are also present. The Wza-Wzb-Wzc complex exports this capsule.

### KL1 in 307-0294 and AYE

KL1 shares region 1 containing *gna* and *gne2* with KL4 and KL9. This configuration is often found in strains that synthesize UDP-D-Gal*p*NAcA e.g. *wbpO-wbpP* from *P. aeruginosa* O6 [38]-[41]. Very recently, a structure was reported for the K1 capsule [24]. It includes D-Gal*p*NAcA and two other sugars, D-Glc*p*NAc6OAc and UDP-2-acetamido-4-(R)-2,4,6-trideoxy-α-D-glucopyranose (D-Qui*p*NAc4NR, where R is acetyl or 3-OH-butyrate). Strain 24 has an identical structure for what is called an O antigen but may be capsule [42]. The D-Glc*p*NAc6OAc can be synthesised via pathways shown in Figure 4, and acetylated by the acetyltransferase encoded by *atr1* in KL1. Genes for the two glycosyltransferases required to form the internal glycosyl linkages of the K1 capsule repeat unit, and the *itrA1* for an initiating transferase are present in KL1. However, the identity of the first sugar is not known.

**Figure 6:**
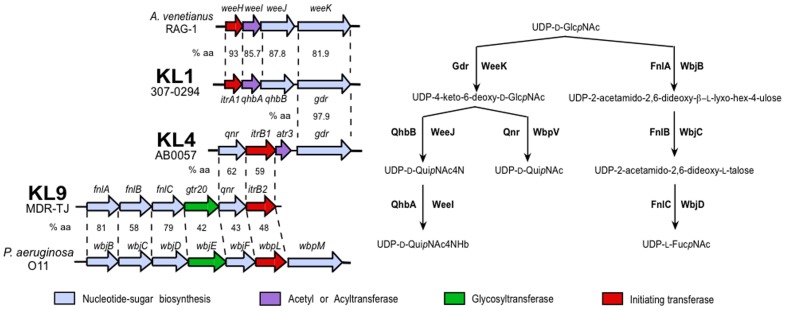
Genes and pathways involved in the synthesis of UDP-D-QuipNAc4NHb, UDP-D-Qui*p*NAc and UDP-L-Fuc*p*NAc. KL1 regions 5 and 6 are compared to a region from the K locus of *A. venetianus* RAG-1 [GenBank accession AJ243431], and to KL4 regions 5 and 6. A region from KL9 is compared to KL4 and a region in the O11 O-antigen locus from *P. aeruginosa* [GenBank accession AF147795]. The biosynthesis pathways of UDP-D-QuipNAc4NHb [23], UDP-L-Fuc*p*NAc [45] and UDP-D-Qui*p*NAc [40], [65] are shown on the right. Horizontal arrows represent genes showing the direction of transcription, with gene names shown above. Genes are coloured by the predicted function of their gene product and the colour scheme is shown the below. The% identity between predicted gene products is shown between aligned pairs of gene clusters. Regions are defined in Figure 3. The figure is drawn to scale from the GenBank entries listed in Table 1 and above.

The KL1 gene cluster also includes *qhbA*, *qhbB* and *gdr* genes for the synthesis of UDP-D-Qui*p*NAc4NR (regions 5 and 6 in Figure 3) located just upstream of *galU*. The gene products share over 80% sequence identity with WeeI, WeeJ and WeeK of *A. venetianus* RAG-1, respectively (Figure 6), that are predicted to synthesise UDP-D-Qui*p*NAc4NR, a derivative of UDP-4-amino-4,6-dideoxy-D-Glc*p*NAc (UDP-D-Qui*p*NAc4N) that is present in the RAG-1 capsule structure [13], [23]. The first step of this pathway involves Gdr (WeeK), a UDP-D-Glc*p*NAc 4,6-dehydratase that generates UDP-4-keto-6-deoxy-D-Glc*p*NAc, an intermediate that also can be utilised in the synthesis of UDP-D-Fuc*p*NAc and UDP-D-Qui*p*NAc [43]. Gdr is 51% identical to WbpM, a known UDP-D-Glc*p*NAc 4,6-dehydratase from *P. aeruginosa* O11 [GenPept accession AAD45269.1]. QhbB and WeeJ are predicted 4-aminotransferases that convert UDP-4-keto-6-deoxy-D-Glc*p*NAc to UDP-D-Qui*p*NAc4N, and QhbA and WeeI are acyltransferases that are predicted to add the acetyl or 3-hydroxybutyryl group as the final step in the synthesis of UDP-D-Qui*p*NAc4NR (Figure 6).

### Additional sugar synthesis operons in KL4 and KL9

The *gdr* gene is also found in KL4 (region 6 in Figure 3), and the gene product is 97.9% identical to the KL1 Gdr. However, KL4 includes a *qnr* gene (region 7 in Figure 3) rather than *qhbA* and *qhbB*. Qnr shares 46% identity with WbpV from *P. aeruginosa* O6 [GenPept accession AAF23991.1], which converts the product of the Gdr reaction to *N*-acetyl-D-quinovosamine (UDP-D-Qui*p*NAc) [44]. Hence, it is likely that AB0057 has D-Qui*p*NAc in its capsule structure. A *qnr* gene (region 7 in Figure 3) is also found in KL8 and KL9. The Qnr of KL8 and KL9 are 98% identical to one another, and 62% identical to Qnr of KL4. However, since *gdr* is not present in KL8 and KL9, it is likely that the capsule of strains carrying KL8 and KL9 will not contain D-Qui*p*NAc.

In KL9, a group of six genes (*fnlABC/gtr18/qnr/itrB2*) is similar to a module found in *P. aeruginosa* O11 [GenBank accession AF147795] that includes genes for WbjB, WbjC and WbjD proteins (see Figure 6), which synthesise *N*-acetyl-L-fucosamine (UDP-L-Fuc*p*NAc) [45]. Homologues of these genes are usually annotated as *fnlA*, *fnlB* and *fnlC,* and are found in the same genetic arrangement in many bacterial species [41], [46]–[48]. The high level of identity between the KL9 Fnl proteins and their Wbj counterparts (see Figure 6) indicates that they are likely to produce UDP-L-Fuc*p*NAc. The same group of genes is found in KL8, and has near identical sequence to KL9. The products of the remaining three genes in the module from *P. aeruginosa* O11 are only 42 to 48% identical to the corresponding products of KL9, and *qnr* and *itrB2* appear to be remnants of a UDP-D-Qui*p*NAc biosynthesis operon as described above.

### Synthesis of a pseudaminic acid relative

In KL2 and KL6, the *psa* genes found adjacent to *gna* in region 3 (Figure 3) predict products that are at least 96.5% identical to one another, and were predicted to direct the synthesis of a pseudaminic acid relative. The predicted products of these genes are similar to products of the *psb* genes of *Shigella boydii* type 7 O antigen (Figure 5B). The Psb proteins synthesise 5-acetamido-3,5,7,9-tetradeoxy-7-[(*R*)-3-hydroxybutanoylamino]-L-*glycero*-L-*manno*–non-2-ulosonic acid, a pseudaminic acid relative, which is seen in the type 7 O antigen [39]. Most Psa enzymes share 57% to 83% sequence identity with the corresponding Psb protein (see Figure 5B). The exceptions are PsaD and PsaE, which are less than 40% identical to Psb4 and Psb5, respectively. Psb5 is predicted to acylate the UDP-2-acetamido-4-amino-2,4,6-trideoxy-β-L-altrose pathway intermediate, and Psb4 then cleaves the UDP linkage from the product of this reaction. The differences observed suggest that PsaE may acylate the pathway intermediate differently, changing in turn the substrate of PsaD, which functions downstream in the pathway. KL2 and KL6 may therefore synthesize a pseudaminic acid relative with a different acylation pattern to the form in the *S. boydii* Type 7 O antigen.

### Synthesis of a legionaminic acid relative

The KL5, KL7 and KL8 loci each contain an *lga* gene module found adjacent to *gna* gene (region 4 in Figure 3). The KL5 and KL7 *lga* gene products are near identical (99%), and share 54 to 99% identity with KL8 Lga proteins. Four of the 7 Lga proteins share more than 50% identity with Elg proteins encoded in the *E. coli* O61 O-antigen gene cluster [GenBank accession GU220362] (see Figure 5C), and the O61 O antigen contains 5,7-diacetamido-3,5,7,9-tetradeoxy-L-*glycero*-D-*galacto*-non-2-ulosonic acid (8eLeg5Ac7Ac), an 8-epimer of legionaminic acid [49]. The *elg* genes and *lga* genes are in a similar order (see Figure 5C). However, the acetyltransferases (LgaE in KL5/KL7 and LgaI in KL8), are only are 56% identical to one another and are not significantly related to the O61 acetyltransferase, Elg5. Hence, there may be a difference in acetylation patterns between the KL5/KL7, KL8 and O61 sugar precursors. Likewise, the synthetase proteins, LgaD in KL5/KL7 and LgaH in KL8, differ in sequence (64% identical) and are only 43% identical to Elg4. This may reflect differences in the substrate specificity of LgaD, LgaH and Elg4 caused by LgaE, LgaI and Elg5 acetylating the same substrate differently. Hence, it is likely that strains carrying KL5, KL7 or KL8 present a form of legionaminic acid, though the position of the acetyl group is unclear.

### Repeat-unit construction

The variable region of each K locus contains a gene predicted to encode an initiating transferase to add the first sugar to the UndP carrier. Two families of initiating transferase enzymes were identified in this set, and the genes, *itrA* and *itrB* (red arrows in Figure 3), code for proteins of different lengths; ItrA are ∼205 aa and ItrB are ∼337 aa. KL8 and KL9 unusually encode two, one of each type, and the genes are located adjacent to each other.

The KL8 and KL9 *itrB2* gene products are near identical (98.5%), whereas ItrB1, encoded by KL4, is only 59% identical to ItrB2. Both ItrB are related to the WbpL_O6_ initiating transferase from the *P. aeruginosa* O6 locus, as ItrB1 and ItrB2 share 49.7% and 45.8% identity with WbpL_O6_, respectively. WbpL_O6_ transfers UDP-D-Qui*p*NAc to UndP [40], and the proximity of *itrB1* to *qnr* and *gdr* in KL4 suggests that UDP-D-Qui*p*NAc may be the substrate for ItrB enzymes and form the first sugar of the repeat unit. The absence of *gdr* in KL8 and KL9, which precludes synthesis of UDP-D-Qui*p*NAc, suggests that ItrB2 is redundant, and the *itrB2* gene is a remnant of a past evolutionary event as described above.

**Figure 7:**
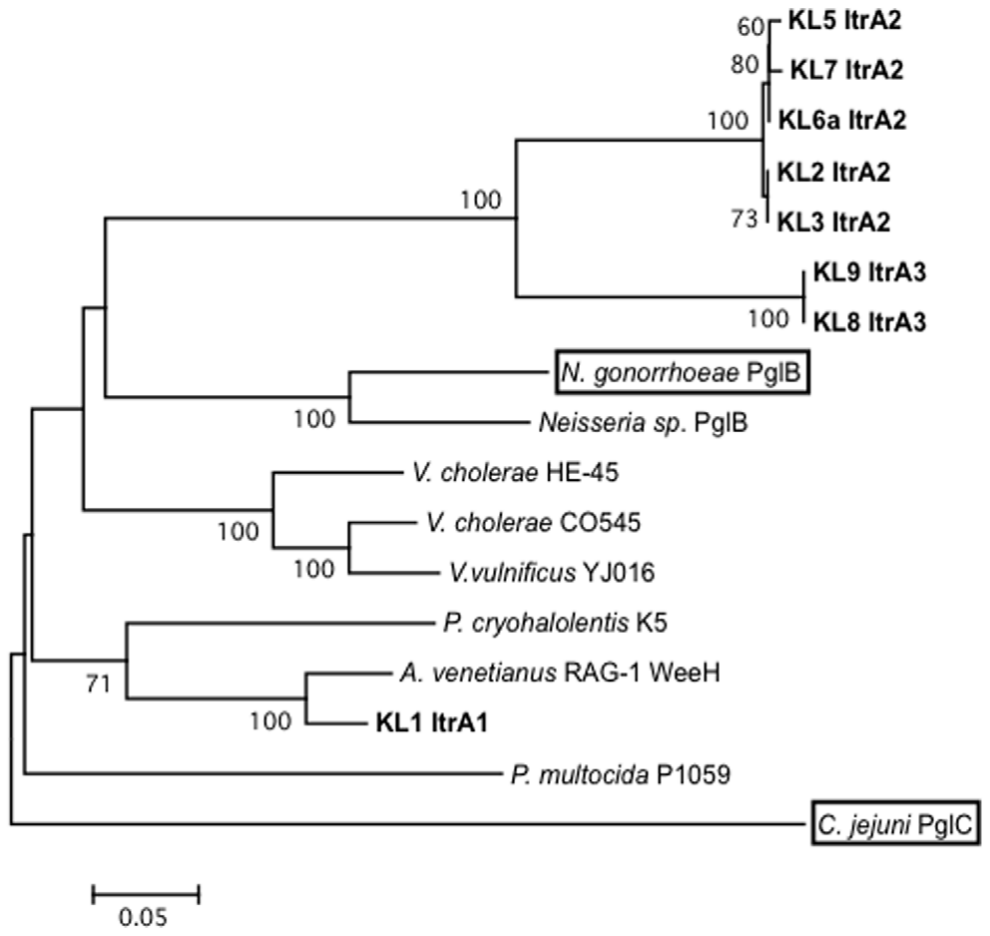
Phylogenetic analysis of initiating glycosyltransferases. Phylogeny of ItrA related protein sequences. *A. baumannii* ItrA are indicated by bold-face type, and ItrA for which biochemistry is available are boxed. Bootstrap values based on 100 replicate trees are shown. Abbreviations and GenPept accessions are as follows: *P. cryohalolentis* K5 is *Psychrobacter cryohalolentis* K5 [YP_579903.1]; *P. multocida* P1059 is *Pasturella multocida* P1059 [EJZ79825]; *V. cholerae* O5 is *Vibrio cholerae* CO545 [ADF80985.1]; *N. gonorrhoeae* PglB is *Neisseria gonorrhoeae* MS11 [ZP_06132416.1]; *Neiserria sp* PglB [EFI22895.1]; *V. vulnificus* YJ016 [NP_933154.1]; *V. cholerae* HE-45 [EJH66138.1]; *C. jejuni* PglC is *Campylobacter jejuni* NCTC 11168 [CAL35241.1]. Both PglB and PglC are specific for UDP-D-Qui*p*NAc4NAc [66], [67].

Three distinct ItrA proteins were identified (Figure 7). The *itrA2* gene is the most common in this set, found in 5 of the 9 loci (Figure 3), and the ItrA2 proteins are very closely related, with pairwise sequence identities between 99 and 100%. As the first sugar of the ATCC 17978 (KL3) pentasaccharide is known to be D-Gal*p*NAc [4], UDP-D-Gal*p*NAc is the likely substrate for ItrA2. ItrA3 (identical in KL8 and KL9) is 76.1% identical to ItrA2, suggesting that ItrA3 may recognise the same or a similar substrate. However, KL1 ItrA1 at 60.8% identity to ItrA2 is more distantly related (see Figure 7). KL1 ItrA1 is 93% identical to WeeH from *A. venetianus* RAG-1 (Figure 6). The repeat unit of RAG-1 is known to contain D-Gal*p*NAc6OAc, D-Gal*p*NAcA and D-Qui*p*NAc4NHb but the identity of the first sugar is not known [50]. Hence, one of these sugars should be the substrate for ItrA1.

Each K locus includes 2 to 4 *gtr* genes for glycosyltransferase enzymes that are required for the assembly of the repeat unit, and the known repeat units include 3–6 sugars (see Table S1). Overall, 22 distinct Gtr were identified when Gtr with greater than 85% aa identity were assigned the same number. The association of specific Gtr with a sugar biosynthesis cluster suggests that that sugar is likely to be one of the substrates. For example, the *gtr20* gene is found only in KL8 and KL9 immediately downstream of the *fnl* operon (Figure 6). Furthermore, Gtr20 is 42% identical to WbjE from *P. aeruginosa* O11, which is specific for UDP-L-Fuc*p*NAc [51].

### Repeat-unit translocation and polymerisation

The repeat units once formed are translocated into the periplasmic space where they are polymerised. Wzx and Wzy inner membrane proteins perform these functions. In most Gram-negative bacteria, Wzx and Wzy typically share low levels of aa identity, making their identification by BLASTp searches difficult. Consequently, they are usually found via the presence of an appropriate number of predicted transmembrane segments, 10–14 for Wzx and 9–12 for Wzy. They are then distinguished from one another using Pfam classifications. In the *A. baumannii* K loci, both *wzx* and *wzy* genes were found in the central variable region, except the *wzy* of KL8 (see Figure 3).

The predicted Wzy proteins from the *A. baumannii* K loci share no significant sequence identity to each other. This includes KL7 and KL5, which otherwise share a large region of DNA identity. Similarly, the Wzx encoded by most of the K loci do not share significant sequence identity. However, the *wzx* genes of KL5, KL7 and KL8, which are all located immediately downstream of the *lga* gene module (region 4 in Figure 3), encode Wzx products that share identities between 79% and 99%. Surprisingly, Wzx of KL6 is also 80–85% identical to Wzx of KL5, KL7 and KL8.

### Other features

In two gene clusters, KL3 and KL4, acyl- or acetyl-transferase genes that could not be associated with a specific sugar biosynthesis pathway were identified. The enzymes encoded by these *atr* genes are likely to modify the final repeat unit. The KL4 gene cluster also contains a putative pyruvate transferase gene, *ptrA*, which could also be involved in repeat unit modification. The product of the *kpsS* gene in KL2 is 30% identical to KpsS from the *E. coli* K5 capsule locus, that is predicted to transfer 3-deoxy-D-*manno*-oct-2-ulosonic *acid* (Kdo) residues onto the capsule oligosaccharide unit, and it is possible that a Kdo is also part of the *A. baumannii* KL2 repeat unit.

### Attachment of capsule to the cell surface

One of the factors that distinguish *E. coli* group 1 capsule gene clusters from group 4 is the presence of a *wzi* gene adjacent to the *wza-wzb-wzc* module [8]. The exact function of Wzi is unknown, however deletion of *wzi* in *E. coli* does not prevent capsule formation but significantly decreases the proportion of cell-bound capsule relative to the amount released into the extracellular environment [52]. A *wzi* gene was not found in any of the *A. baumannii* K loci. However, a gene [locus tag AB57_1078 in CP001182] encoding a homologue of Wzi from *E. coli* O9a:K30 [GenPept accession AAD2156.1] (48% identity, 63% similarity) was found between *lysS* and *cysD* (see Figure 1 for the location of *wzi*).

## Discussion

In this study, we examined whether *A. baumannii* produces an O antigen, capsule or both. This question has long been debated, and is reviewed in [9]. Many biochemical studies have reported difficulty in determining the origin of the isolated polysaccharide [53]–[56]. However, there is clear evidence for the production of a capsule in *A. baumannii* [3], [10], [24] and in other *Acinetobacter* sp., where it is sometimes also called emulsan [25], [57]. As there is only one locus in the genomes that could produce this capsule, it was named the K locus. Our conclusion that that an LPS is not produced in *A. baumannii*, is supported by finding a gene encoding a WaaL homologue in the vicinity of the OC locus in two genomes of non- *baumannii Acinetobacter* species [GenPept accession numbers ZP_09142563.1 and ZP_10859085.1]. However, our conclusion requires rigorous biochemical confirmation. In particular, evidence that the PglL *O*-oligosaccharyltransferase, which adds a single oligosaccharide repeat unit to certain proteins [4], cannot ligate the polysaccharide to the LOS moiety would be valuable. However, in most Gram-negative species the PglL and WaaL exhibit strict specificity for proteins or LOS, respectively [30]. The possibility that there is a ligase which cannot be detected using the bioinformatic searches undertaken here can only be excluded when there is clear biochemical evidence that no LPS is present.

We examined in detail two regions of significant variability in the *A. baumannii* genomes and, using published information on the structure of the OC and of capsule repeat units, were able to associate the OC locus with synthesis of the OC component of the LOS and the K locus with synthesis of capsule. In Gram-negative bacteria, polysaccharide biosynthesis loci are typically hotspots of genomic variability, and with only 10 genomes yielding 9 K loci and 3 OC loci, it is clear that this will also apply to *Acinetobacter*. Indeed, our analysis of available draft genomes has identified many more K-locus configurations and several more OC-locus configurations (J. J. Kenyon and R. M. Hall, unpublished). However, a novel feature of the *Acinetobacter* K loci is the presence of module B which included genes for the synthesis of simple UDP-linked sugars that in other Gram-negative bacteria are usually scattered around the genome. Furthermore, the outermost part of module B is conserved at levels close to those for other conserved parts of the genome.

The presence of two different K loci in GC1 strains has been noted previously [18]. However, examination of the OC loci revealed that it also varied with AB0057 carrying OCL3 (see Table 2). The 5 GC2 strains listed in Table 2 each carried a different K locus, though the OC locus was conserved. An 11th complete *A. baumannii* genome and 6th complete GC2 genome for strain TYTH-1 [58] was released after this work was completed. It includes OCL1 and a further different K locus (KL10) that contains a gene module for the synthesis of a 3-acetamido-3,6-dideoxy-D-galactose (D-Fuc3NAc) like sugar structure (see Figure S1). This variation was foreshadowed by MLST typing [1]. The *gpi* gene, which is included in the Oxford MLST scheme for the differentiation of *A. baumannii* strains [33], is located in module B, and hence in a region that is significantly more variable than most housekeeping genes. Indeed, a different *gpi* allele is found in each gene cluster type, and this suggests that the *gpi* sequence could be used as an indication of the K-locus type present.

This is the first study to undertake a detailed analysis of the loci that direct the synthesis of surface polysaccharides in the clinically important pathogen, *A. baumannii,* and predict the role of each of the genes. The diversity noted in both the K and OC loci, particularly variation within individual clonal complexes, will further our understanding of this species by increasing our understanding of the evolution of clonal lineages. It will also enable the development of a combined PCR typing scheme for both the K and OC loci which, used together with other available typing tools, could simplify epidemiological studies. The information provided by these annotations will also facilitate much-needed biochemical studies on the surface polysaccharides. Exchange of the loci described here is occurring quite frequently, and more detailed analysis of other parts of the genomes in each of the global clones should provide insight into when these changes occurred relative to the changes that resulted in the acquisition of antibiotic resistance genes.

## Materials and Methods

### Bioinformatic analysis

The two variable regions previously reported [18], [21] were extracted from the completed genomes available in GenBank as of 30^th^ September, 2012 [see Table 1 for GenBank accession numbers]. Each region was assessed for ORFs using the NCBI ORF finder tool (ORF Finder (Open Reading Frame Finder) website.http://www.ncbi.nlm.nih.gov/projects/gorf/. Accessed 22 February 2013). BLASTp [59] similarity searches and Pfam predictions [60] were used to characterise predicted protein sequences. These sequences were also assessed for predicted transmembrane segments using the TMHMM 2.0 program (Prediction of transmembrane helices in proteins website. Available: http://www.cbs.dtu.dk/services/TMHMM-2.0/. Accessed 22 February 2013). IS elements were identified using the IS Finder database (IS Finder website. Available: https://www-is.biotoul.fr//is.html. Accessed 22 February 2013).

Multiple alignments and pairwise sequence comparisons were carried out using ClustalW [61] and Matcher programs.Phylogenetic trees were constructed using ClustalW pairwise alignments and the neighbour joining tree method using MEGA5 software [62]. Bootstraps were calculated based on 100 tree replicates.

The BLASTp search for predicted proteins with similarity to known WaaL O-antigen ligases, used query protein sequences of WaaL from *P. aeruginosa* PAO1 [GenPept accession NP_253686] and from each of the 5 defined *E. coli* core types K12, R1, R2, R3 and R4 [GenPept accessions NP_418079, AAC69671, AAC69648, AAC69661, and AAC69682 respectively]. The BLASTp search for predicted proteins with similarity to Wzi, used the *E. coli* O9a:K30 Wzi protein as the query sequence [GenPept accession AAD2156.1].

### Correction of frameshifts

Regions containing possible frameshifts in AB0057 and ATCC17978 were resequenced. PCR amplification were carried out as described previously [63] using genomic DNA as template and primers specific for these regions (Integrated DNA Technologies, Inc., San Diego). PCR products were purified as described previously [63], and sequenced using an AB3730*xl* sequencing platform at the Australian Genome Research Foundation, Sydney, Australia. All frameshifts identified in the K loci of these strains were corrected prior to analysis.

### Annotation of genes in K and OC loci

An annotation scheme was developed to distinguish genes by the function of their gene product. All gene identifiers used in this annotation scheme are summarised in Table 3. Briefly, genes predicting proteins from well-defined families were assigned names that are used for most bacterial species [26]. The genes for specific sugar biosynthesis pathways were assigned a prefix identifying the final sugar product and an alphabetical identifier by order of their position in the locus. The *itr* gene products were further separated into Itr families A or B. Numbers were assigned to differentiate sequence types of *atr*, *gtr* and *itr* genes, if the aa sequence identity of the gene products was greater than 85%.

## Supporting Information

Figure S1 **Arrangement and preliminary annotation of the KL10 capsule gene cluster of** ***A. baumannii*** **TYTH-1**. K-locus name with strain name beneath is indicated on the left. Horizontal arrows represent genes showing the direction of transcription, with assigned gene names shown above. Genes are coloured by the predicted functional group of their gene product with the colour scheme shown in Figure 3. The figure is drawn to scale.
(DOCX)

Table S1 Annotations for the PglL *O*-oligosaccharyltransferase identified by Iwashkiw *et al.* (2012)
(DOCX)

Table S2 Sugar composition of known *A. baumannii* oligosaccharide structures.
(DOCX)
