# Supplementary material for: Variation in the Complex Carbohydrate Biosynthesis Loci of Acinetobacter baumannii Genomes
Source: PLoS One. 2013 Apr 16;8(4):e62160. doi: 10.1371/journal.pone.0062160 (PMC3628348; doi:10.1371/journal.pone.0062160)
Supplement: Figure S1 — Arrangement and preliminary annotation of the KL10 capsule gene cluster of A. baumannii TYTH-1. K-locus name with strain name beneath is indicated on the left. Horizontal arrows represent genes showing the direction of transcription, with assigned gene names shown above. Genes are coloured by the predicted functional group of their gene product with the colour scheme shown in Figure 3. The figure is drawn to scale. (DOCX) [file pone.0062160.s001.docx]

**Figure S1. Arrangement and preliminary annotation of the KL10 capsule gene cluster of *A. baumannii* TYTH-1**. K-locus name with strain name beneath is indicated on the left. Horizontal arrows represent genes showing the direction of transcription, with assigned gene names shown above. Genes are coloured by the predicted functional group of their gene product with the colour scheme shown in Figure 3. The figure is drawn to scale.
