# Supplementary material for: Variation in the Complex Carbohydrate Biosynthesis Loci of Acinetobacter baumannii Genomes
Source: PLoS One. 2013 Apr 16;8(4):e62160. doi: 10.1371/journal.pone.0062160 (PMC3628348; doi:10.1371/journal.pone.0062160)
Supplement: Table S1 — Annotations for the PglL O-oligosaccharyltransferase identified by Iwashkiw et al. (2012) (DOCX) [file pone.0062160.s002.docx]

**Table S1**: Annotations for the PglL *O*-oligosaccharyltransferase identified by Iwashkiw *et al.* (2012)^a^

| **STRAIN** | **ACCESSION** | **GenPept ANNOTATION** | **BLASTp identity to PglL (A1S_3176)** |
| --- | --- | --- | --- |
| ***Acinetobacter* *baumannii*** |  |  |  |
| **ATCC 17978** | **YP_001086175.1** | **hypothetical protein A1S_3176** | **100%** |
| AYE | YP_001712289.1 | hypothetical protein ABAYE0306 | 82% |
| 307-0294 | YP_002324267.1 | O-Antigen polymerase family protein | 82% |
| ACICU | YP_001848035.1 | Lipid A core - O-antigen ligase | 91% |
| AB0057 | YP_002320930.1 | O-antigen polymerase family | 81% |
| SDF | YP_001705998.1 | hypothetical protein ABSDF0316 | 91% |
| 1656-2 | YP_005515999.1 | Lipid A core--O-antigen ligase | 91% |
| TCDC-AB0715 | YP_005800465.1 | Lipid A core--O-antigen ligase | 91% |
| MDR-ZJ06 | YP_005527519.1 | Lipid A core--O-antigen ligase | 91% |
| MDR-TJ | YP_006288269.1 | Lipid A core--O-antigen ligase-like protein | 91% |

**The red indicates experimental evidence is available for this strain.**

^a^ The *pglL* gene is located in the Type 4 pilin locus (see Figure 1).
