# Supplementary material for: Variation in the Complex Carbohydrate Biosynthesis Loci of Acinetobacter baumannii Genomes
Source: PLoS One. 2013 Apr 16;8(4):e62160. doi: 10.1371/journal.pone.0062160 (PMC3628348; doi:10.1371/journal.pone.0062160)
Supplement: Table S2 — Sugar composition of known A. baumannii oligosaccharide structures. (DOCX) [file pone.0062160.s003.docx]

**Table S2.** Sugar composition of known *A. baumannii* oligosaccharide structures.

| **Strain^a^** | **Simple sugars** | **Other sugars** | **No. Gtr required^b^** | **Reference** |
| --- | --- | --- | --- | --- |
| 307-0294^c^ | D-Glc*p*NAc6Ac  D-Gal*p*NAcA | D-Qui*p*NAc4NR^d^ | 2 | [[1](#_ENREF_1)] |
| 24 | D-Glc*p*NAc6Ac  D-Gal*p*NAcA | D-Qui*p*NAc4NR^d^ | 2 | [[2](#_ENREF_2)] |
| MG1^c^ | D-Glc*p*NAc | L-Gal*p*NAcA  D-Qui*p*NAc4NR^d^ | 2 | [[3](#_ENREF_3)] |
| SMAL^c^ | D-Glc*p*  D-Gal*p*  D-Glc*p*NAc  D-Gal*p*NAc | D-Glc*p*NAc3NAcA | 4 | [[3](#_ENREF_3)] |
| O1 | D-Gal*p*  D-Glc*p*NAc  D-Gal*p*NAc |  | 2 | [[4](#_ENREF_4)] |
| O2 | D-Gal*p*  D-Gal*p*NAc | D-Fuc*p*3NHb | 5 | [[5](#_ENREF_5)] |
| O5 | D-Glc*p*NAc  D-Gal*p*NAcA | L-Fuc*p*NAc | 3 | [[6](#_ENREF_6)] |
| O7 | D-Glc*p*NAc | L-Rha*p* | 5 | [[7](#_ENREF_7)] |
| O10 | D-Glc*p*NAc | D-Man*p*NAc  L-Rha*p* | 4 | [[8](#_ENREF_8)] |
| O11 | D-Glc*p*  D-Gal*p*  D-Gal*p*NAc |  | 4 | [[9](#_ENREF_9)] |
| O12 | D-Glc*p*  D-Glc*p*NAc  D-Gal*p*NAc |  | 3 | [[10](#_ENREF_10)] |
| O16 | D-Glc*p*  D-Glc*p*NAc  D-Gal*p*NAc |  | 3 | [[11](#_ENREF_11)] |
| O18 | D-Gal*p*  D-Gal*p*NAc | D-Man*p*NAc | 3 | [[12](#_ENREF_12)] |
| O23 | D-Gal*p*  D-Glc*p*NAc  D-Gal*p*NAc | D-Qui*p*3NHb | 4 | [[10](#_ENREF_10)] |
| O24 | D-Glc*p*NAc | Legionaminic acid relative  L-Fuc*p*NAc | 3 | [[13](#_ENREF_13)] |
| 9 | D-Glc*p*  D-Gal*p*NAc | D-Qui*p*4NAc | 4 | [[14](#_ENREF_14)] |
| 34 | D-Gal*p*  D-Gal*p*NAc | D-Fuc*p*3NBuOH | 5 | [[15](#_ENREF_15)] |
| 214 (O22) | D-Glc*p*  D-Gal*p*  D-Gal*p*NAc |  | 2 | [[16](#_ENREF_16)] |

^a^ Strains with names beginning with O are representatives of that designated ‘O type’ in the antigenic typing scheme.

^b^ Maximum number of glycosyltransferases needed for number of linkages without the Itr

^c^ The structures of 307-0294, SMAL and MG1 are known capsules. ATCC 17961 [[17](#_ENREF_17)] has the same structure as SMAL. The structure for the ATCC 17978 protein glycan [[18](#_ENREF_18)] is also identical to the SMAL structure.

^d^ R is Ac (acetyl) or Hb (3-OH-butyrate) group

**References**

1. Russo T, Beanan J, Olson R, MacDonald U, Cox A, et al. (2013) The K1 capsular polysaccharide from *Acinetobacter baumannii* is a potential therapeutic target via passive immunization. Infect Immun.

2. Vinogradov E, Brade L, Brade H, Holst O (2003) Structural and serological characterisation of the O-antigenic polysaccharide of the lipopolysaccharide from *Acinetobacter baumannii* strain 24. Carbohydr Res 338: 2751-2756.

3. Fregolino E, Gargiulo V, Lanzetta R, Parrilli M, Holst O, et al. (2011) Identification and structural determination of the capsular polysaccharides from two *Acinetobacter baumannii* clinical isolates, MG1 and SMAL. Carbohydr Res 346: 973-977.

4. Galbraith L, Sharples J, Wilkinson S (1999) Structure of the O-specific polysaccharide for *Acinetobacter baumannii* serogroup O1. Carbohydr Res 319: 204-208.

5. Haseley S, Wilkinson S (1995) Structural studies of the putative O-specific polysaccharide of *Acinetobacter baumannii* O2 containing 3,6-dideoxy-3-*N*-(D-3-hydroxybutyryl)amino-D-galactose. Eur J Biochem 233: 899-906.

6. Haseley S, Wilkinson S (1996) Structure of the O-specific polysaccharide of *Acinetobacter baumannii* O5 containing 2-acetamido-2-deoxy-D-galacturonic acid. Eur J Biochem 237: 229-233.

7. Haseley S, Wilkinson S (1998) Structure of the O-7 antigen from *Acinetobacter baumannii*. Carbohydr Res 306: 257-263.

8. Haseley S, Wilkinson S (1994) Structure of the putative O10 antigen from *Acinetobacter baumannii*. Carbohydr Res 264: 73-81.

9. Haseley S, Wilkinson S (1996) Structural studies of the putative O-specific polysaccharide of *Acinetobacter baumannii* O11. Eur J Biochem 237: 266-271.

10. Haseley S, Traub W, Wilkinson S (1997) Structures of polymeric products isolated from the lipopolysaccharides of reference strains for *Acinetobacter baumannii* O23 and O12. Eur J Biochem 244: 147-154.

11. Haseley S, Diggle H, Wilkinson S (1996) Structure of a surface polysaccharide from *Acinetobacter baumannii* O16. Carbohydr Res 293: 259-265.

12. Haseley S, Wilkinson S (1997) Structure of the O18 antigen from *Acinetobacter baumannii*. Carbohydr Res 301: 187-192.

13. Haseley S, Wilkinson S (1997) Structural studies of the putative O-specific polysaccharide of *Acinetobacter baumannii* O24 containing 5,7-diamino-3,5,7,9-tetradeoxy-L-*glycero*-D-*galacto-*nonulosonic acid. Eur J Biochem 250: 617-623.

14. Haseley S, Holst O, Brade H (1998) Structural studies of the O-antigen isolated from the phenol-soluble lipopolysaccharide of *Acinetobacter baumannii* (DNA group 2) strain 9. Eur J Biochem 251: 189-194.

15. Vinogradov E, Pantophlet R, Dijkshoorn L, Brade L, Holst O, et al. (1996) Structural and serological characterisation of two O-specific polysaccharides of *Acinetobacter*. Eur J Biochem 239: 602-610.

16. Haseley S, Galbraith L, Wilkinson S (1994) Structure of a surface polysaccharide from *Acinetobacter baumannii* strain 214. Carbohydr Res 258: 199-206.

17. MacLean L, Perry M, Chen W, Vinogradov E (2008) The structure fo the polysaccharide O-chain of the LPS from *Acinetobacter baumannii* strain ATCC 17961. Carbohydr Res 344: 474-478.

18. Iwashkiw J, Seper A, Weber B, Scott N, Vinogradov E, et al. (2012) Identification of a general *O*-linked protein glycosylation system in *Acinetobacter baumannii* and its role in virulence and biofilm formation. PLoS Pathog 8.
